# The Use of the Goldfinger Dissector (GD) in Laparoscopic Sacrocolpopexy

**DOI:** 10.3389/fmed.2018.00155

**Published:** 2018-05-31

**Authors:** Pascal Talla, Maria Ekotomati, Tara O'Leary, Nordine Ben Ali

**Affiliations:** ^1^Department of Gynecology and Obstetrics, Hospital Cantonal of Neuchâtel, Neuchâtel, Switzerland; ^2^Department of Gynecology and Obstetrics, Hospital Cantonal of Fribourg, Fribourg, Switzerland

**Keywords:** Goldfinger dissector, sacrocolpopexy, genital prolapse, laparoscopy, peritoneal dissection

## Abstract

We tested the employment of the GD to create a retroperitoneal tunnel between the promontory and the vaginal apex during the laparoscopic sacrocolpopexy with a mesh. Thus far no report has experimented the use of the GD in this indication. This study's aim was to evaluate the safety and the interest to use this laparoscopic instrument. Sixteen consecutive patients underwent a laparoscopic sacrocolpopexy with the use of the GD and were compared with a control group constituted by the previous 30 cases. The median operating time was 180 min with the use of the GD and represent a gain of time of 22 min in comparaison with our control group. No conversion to open or complications were recorded. In our limited experience, the use of the GD allows a significant gain of time and limits the amount of peritoneal dissection.

## Introduction

Sacrocolpopexy nowadays constitutes the most effective choice for pelvic prolapse repair, with objective cure achieved in 91% of patients after a mean follow-up of 26 months (1).

Though initially performed by laparotomy, laparoscopic access has proved to be a practicable approach with shorter hospital stays. However the laparoscopic techniques are more time consuming and thus would benefit from a simplification of the mesh insertion procedure.

In our efforts to simplify the procedure we have tested the GD permitting a substantial gain in operative time and possibly less invasive dissection.

**Figure 1:**
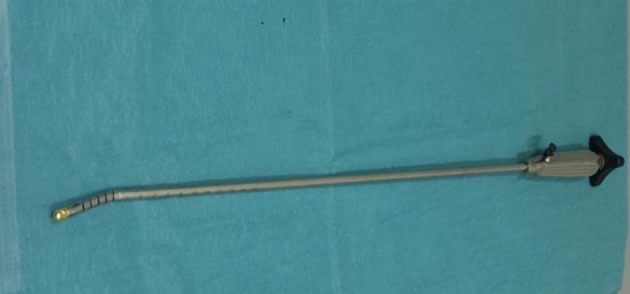
The Goldfinger dissector.

**Figure 2:**
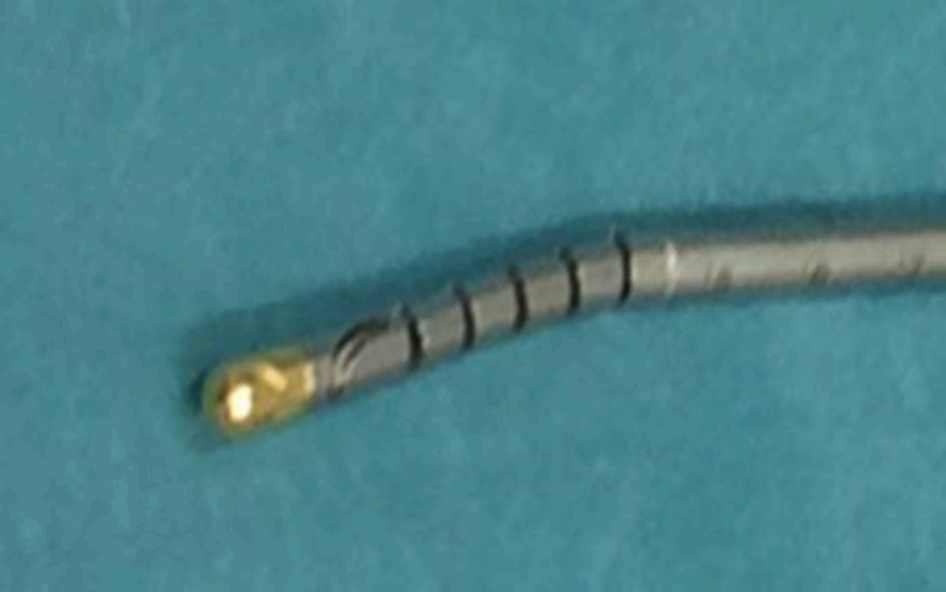
The tip of the Golfinger dissector presenting a chink.

The GD is an endoscopic instrument initially developed as a gastric band introducer in laparoscopic bariatric surgery (2). The GD was then used in distal pancreatectomy (3) and laparoscopic hepatectomy (4). The tip can be angulated with ease over 90° and has a perforated end for tape or thread insertion (Figures 1, 2).

In this paper we describe the feasibility of the use of the GD for retroperitoneal tunneling for mesh sacrocolpopexies.

## Materials and methods

Ethical approval was sought from and granted by the local research ethics committee, which deemed that full ethical approval was not required because the project was considered to be solely a service evaluation. All patients presented with a symptomatic vault prolapse, with at least stage II prolapse of the apex or upper vaginal wall based on the Pelvic Organ Prolapse Quantification System, and gave their written consent for the planned surgical intervention.

Between September 2016 and April 2017, a total of 16 laparoscopic sacrocolpopexies were performed by one unique laparoscopic uro-gynecologic surgeon in one surgical center.

**Figure 3:**
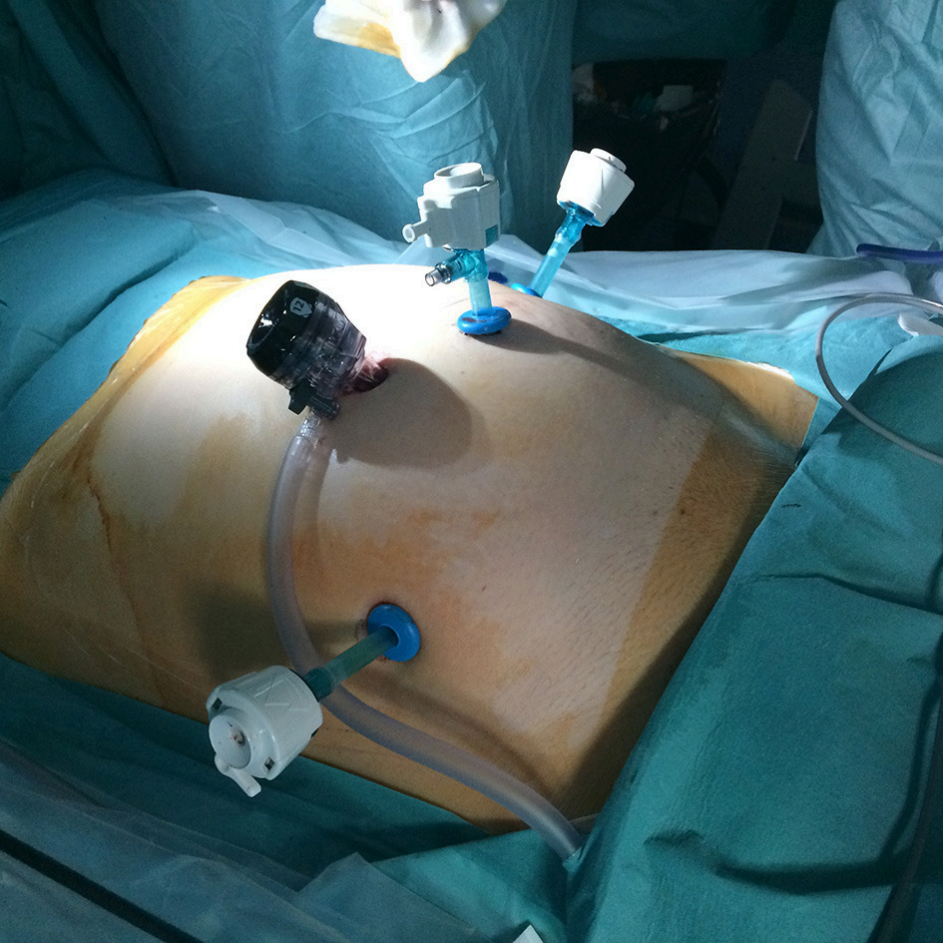
Placements of the trocars.

Four ports are required (Figure 3): One 10 mm umbilical port, two 5 mm ports lateral to the rectus abdominis muscle and medial to the anterior superior iliac spine, and one 5 mm port at the intersection of the umbilical and mammary line. This last port is used as the access port for the GD.

**Figure 4:**
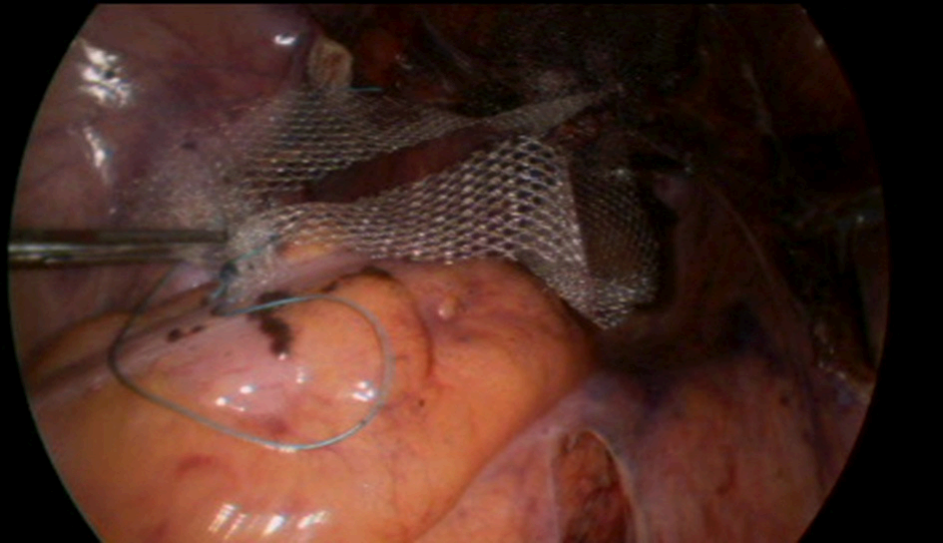
A thread is sutured to the promontery's part of the mesh. The distal part of the mesh is sutured to the anterior and posterior walls oh the vagina.

**Figure 5:**
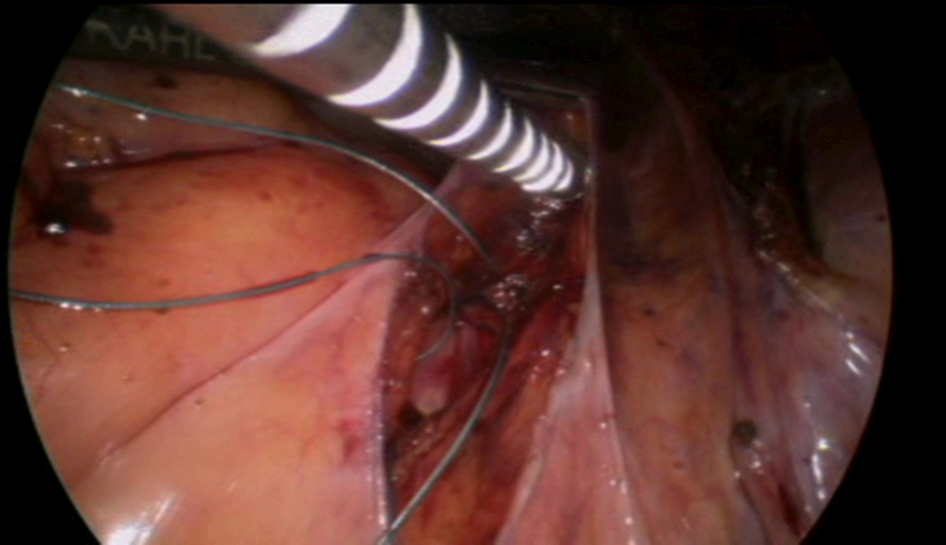
Introduction of the GD.

**Figure 6:**
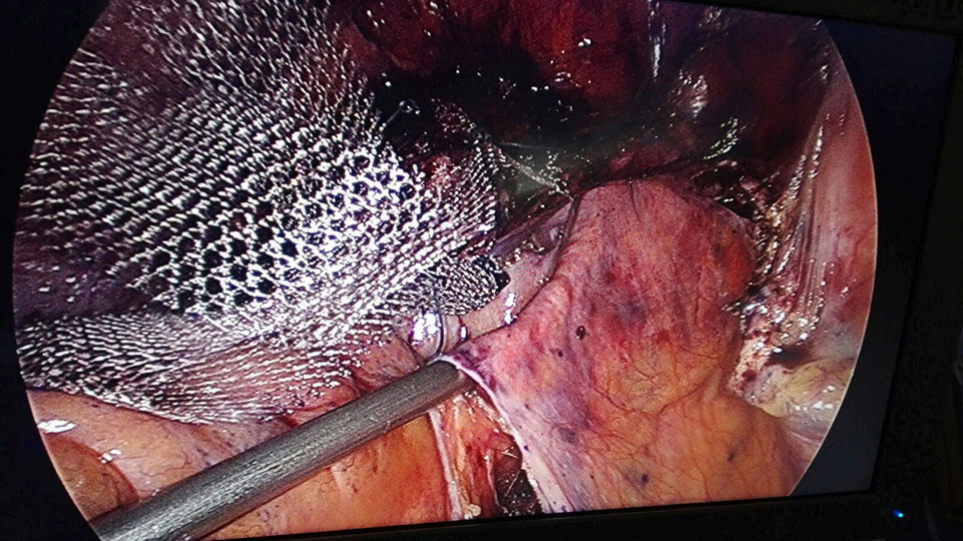
Subperitoneal GD's progression under visual control.

**Figure 7:**
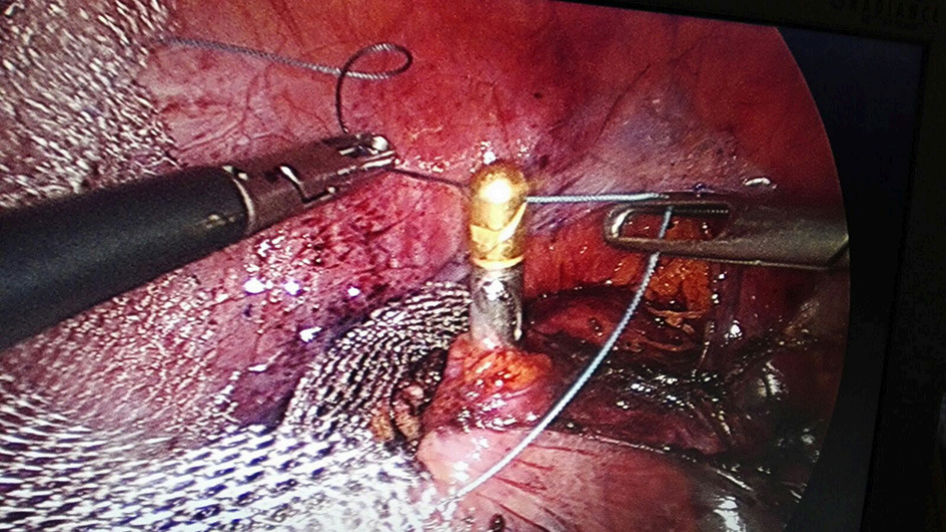
Setting the thread in the GD's chink.

**Figure 8:**
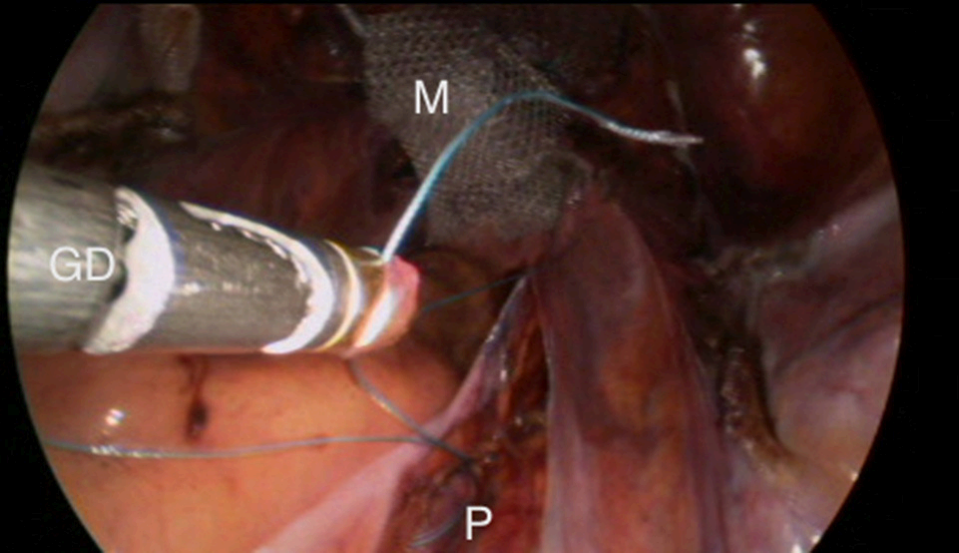
Dragging up the mesh.

**Figure 9:**
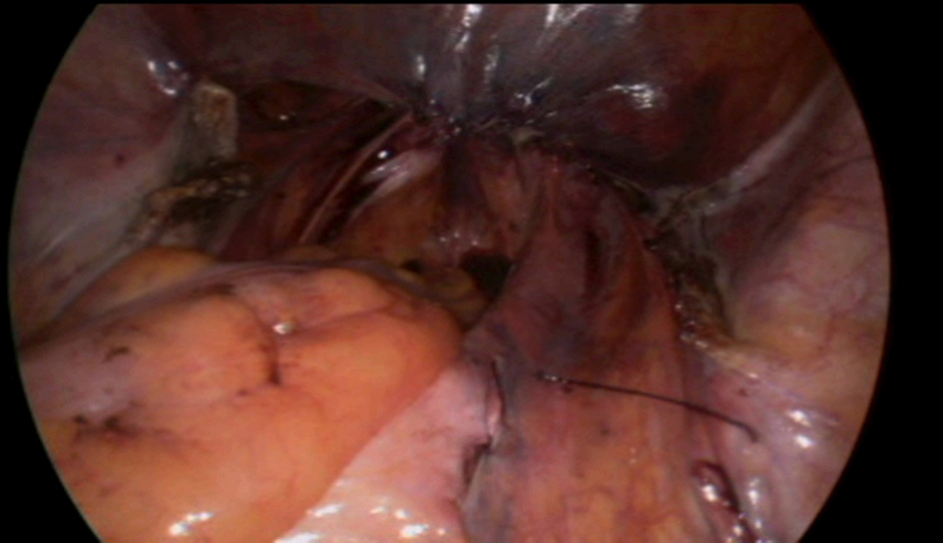
Final result.

Before the introduction of the GD in September 2016, the surgical technique consisted of retroperitoneal dissection from the promontory to the Douglas cul-de-sac along the rectosigmoid colon. This step has been replaced by limited dissection of the peritoneum on the promontory and the cul-de-sac of Douglas. The mesh (large-pore polypropylene) is sutured to the anterior and posterior wall of the vagina. The GD is then inserted through the opening over the promontory and pushed retroperitoneally to the incision in the cul-de-sac, the mesh is attached to the tip and dragged retroperitoneally up to the promontory (Figures 4–9). All the manupulation of the GD is done under visual control.

We created a control group of the previous 30 cases, where the same surgeon performed the sacrocolpopexy using the extended peritoneal incision from the promontory to the cul-de-sac. The characteristics in both groups are comparable with no significant differences (Table 1). The sacrocolpopexy was combined with a subtotal hysterectomy in five patients in the experimental participants and nine patients in the control participant based on the patient preference or a benign gynecologic pathology (fibroïds, dysmenorrhea, or menorrhagea).

**Table 1:** Baseline characteristics of women in the experimental group and control group.

**Characteristic**	**Control group (*n* = 30)**	**Experimental group(*n* = 16)**
Age (years)	54.6 ± 15.1	56.1 ± 14.2
Body mass index (kg/m^2^)	26.6 ± 5.0	27.1 ± 4.4
Previous hysterectomy	10	5
Previous vaginal prolapsus repair	9	5

## Results

The retroperitoneal tunneling technique was used for 16 patients. The median operating time was of 180 min, with a range from 150 to 256 min. No conversion to open was recorded. No peri-operative complications were recorded. During a median post-operative hospital stay of 3 days (ranging from 3 to 4 days) no complications occurred.

In the control group the median operating time was 220 min, representing a median decrease in operating time of 22 min.

## Discussion

After the suture of the mesh to the promontory, most surgeons close the peritoneum over the mesh in an attempt to prevent bowel adhesion and internal hernia. In 2005 Elneil et al. published a unique case series of 128 patients undergoing abdominal or laparoscopic sacrocolpopexy without burial of the mesh and didn't report any bowel complications during a median follow-up of 19 months. However this trial is limited and most surgeons continue to encourage peritoneal closure (5).

There are several advantages offered by the Goldfinger dissector: reduced operating time, potentially reduced denervation with less post-operative urinary or bowel dysfunction.

The described technique represents a simplification of the traditional operative technique, allowing a shorter learning curve for new surgeons. In our experience, the blunt tip of the dissector enables retroperitoneal tunneling without hemorrhage. In case of hemorrhage during the use of the GD, we recommand the opening of the peritoneum to permit the direct electrocoagulation of the bleeding vessel(s).

Gokhan and co is conducting a prospective single blind and randomized study on the impact of tunneling during minimally invasive sacrocolpopexy (6). This study will evaluate the effect of not incising the peritoneum on the post-operative urinary and gastro-intestinal symptoms during the first year. The study completion date is estimated for September 2017.

## Conclusion

The use of the Goldfinger Dissector is safe, effective and reproducible and represents a substantial gain of time for laparoscopic sacrocolpopexy.

## Author contributions

PT principal surgical operator. NB article redactor and data collection. ME data collection and iconograpghy treament. TO lecture and correction of the article.

### Conflict of interest statement

The authors declare that the research was conducted in the absence of any commercial or financial relationships that could be construed as a potential conflict of interest.
